# Associations between Maternal and Fetal Levels of Total Adiponectin, High Molecular Weight Adiponectin, Selected Somatomedins, and Birth Weight of Infants of Smoking and Non-Smoking Mothers

**DOI:** 10.3390/ijerph17134781

**Published:** 2020-07-03

**Authors:** Magdalena Chełchowska, Joanna Gajewska, Tomasz M. Maciejewski, Joanna Mazur, Mariusz Ołtarzewski, Jadwiga Ambroszkiewicz

**Affiliations:** 1 Department of Screening Tests and Metabolic Diagnostics, Institute of Mother and Child, Kasprzaka 17a, 01-211 Warsaw, Poland; joanna.gajewska@imid.med.pl (J.G.); mariusz.oltarzewski@imid.med.pl (M.O.); jadwiga.ambroszkiewicz@imid.med.pl (J.A.); 2 Clinic of Obstetrics and Gynaecology, Institute of Mother and Child, Kasprzaka 17a, 01-211 Warsaw, Poland; dyrektor.naczelny@imid.med.pl; 3 Department of Humanization in Medicine and Sexology, Collegium Medicum University of Zielona Góra and Institute of Mother and Child in Warsaw, 65-729 Zielona Góra, Poland; joanna.mazur@imid.med.pl

**Keywords:** tobacco smoke, pregnancy, cord blood, high molecular weight adiponectin, insulin-like growth factor binding protein-1, birth weight

## Abstract

The aim of the study was to determine the relationships between maternal smoking, total adiponectin, high molecular weight adiponectin (HMW adiponectin), selected somatomedins, and the birth weight of newborns. A total of 78 women with a healthy, singleton pregnancy, 41 active smokers and 37 non-smokers, and their offspring were studied. Total and HMW adiponectin, insulin-like growth factor I (IGF-I), and insulin-like growth factor binding protein-1 (IGFBP-1) and 2 (IGFBP-2) were determined in maternal and cord blood by enzyme-link immunosorbent assay. Serum levels of total and HMW adiponectin were lower in smokers compared to the tobacco abstinent in both the mothers (*p* = 0.013; *p* = 0.006) and the infants (*p* = 0.001; *p* = 0.047). In smoking women and their children, serum concentrations of IGF-I were significantly lower (p = 0.014; p = 0.042), IGFBP-1 significantly higher (*p* = 0.009; *p* = 0.039), and IGFBP-2 did not differ from that observed in the non-smoking group. In multivariate analysis performed on the whole group of mothers, the highest impact of serum cotinine and IGFBP-2 levels were indicated for adiponectin and cotinine and the number of cigarettes/day for HMW adiponectin concentration. In correlation analysis estimated separately for smokers and non-smokers, neonatal birth weight was positively associated with total and HMW adiponectin concentrations in umbilical cord blood. Birth weight was also inversely associated with IGFBP-1 and positively correlated with IGF-I levels in maternal serum as well as in cord blood (r = −0.317, *p* = 0.005; r = −0.294, *p* = 0.004; r = 0.245, *p* = 0.031; r = 0.271, *p* = 0.009, respectively). The present study showed the levels of total and HMW adiponectin in umbilical cord blood may have a significant effect on fetal development. Both IGF-I and IGFBP-1 concentrations also play an essential role in fetal growth, which is an important predictor of birth weight. Cigarette smoking during pregnancy negatively affected adiponectin and the insulin growth factor profile in the serum of women and the cord blood and may be the reason for the lower birth weight of the smokers newborns compared with the nonsmokers offspring.

## 1. Introduction

In recent years, the role of adipose tissue markers in intrauterine fetal development has been widely discussed. Adiponectin belonging to the adipocytokine family is a polypeptide hormone that actively participates in the sensitization of peripheral tissues to insulin. In serum, adiponectin circulates in three different isoforms: trimeric (low molecular weight LMW), hexameric medium molecular weight (MMW), and oligomeric high molecular weight (HMW). HMW adiponectin appears to be an active form and the HMW/total adiponectin ratio seems to provide a better reflection of peripheral insulin sensitivity than total adiponectin alone [1]. It is also documented that the proportion of maternal serum adiponectin in HMW form is negatively related with infant birth weight [2,3]. Adiponectin stimulating the metabolic pathways of insulin action is an important factor for regulating the availability of this hormone and glucose, as well as insulin-dependent growth factors to the fetus [1].

The insulin-like growth factor system (somatomedins) is considered to be involved in many aspects of placental development and fetal growth regulation. The structural similarity of insulin-like growth factor I (IGF-I) with insulin and its proven hypoglycemic effect regulated by insulin-like growth factor binding proteins (IGFBP) suggests the participation of this ligand in the regulation of glucose metabolism in both the mother and the fetus. IGF-I is expressed in several fetal tissues, where it stimulates cell growth through endocrine and paracrine effects. The bioavailability of insulin-like growth factors is modulated by a family of six IGF-binding proteins (IGFBPs). Although IGFBP-3 has the highest affinity for IGF-I (about 80%), during gestation, this affinity decreases due to increased protease activity. In pregnancy, IGFBP-1 is the predominant binding protein for IGFs, and its affinity toward IGF-I is three times higher than proteolyzed IGFBP-3 [4]. IGFBP-2 is another binding protein that affects glucose homoeostasis and may inhibit insulin-like growth factor activity [5]. It is assumed that birth weight correlates positively with IGF-I levels and negatively with IGFBP-1 and IGFBP-2 concentrations, but these are not conclusive data [6]. Animal studies conducted in knockout mice showed that IIGF-I and IGF-II are required for optimal fetal and placental growth [7]. Whereas, the significant role of IGFBP-1 in placental growth and morphogenesis in IGFBP-1 transgenic mice has been documented by Crossey et al. [8].

Birth weight, which is an important indicator of fetal well-being, depends on the interaction of many factors of genetic, hormonal, maternal, fetal, and environmental origin. One of the environmental factors that increases the risk of lower birth weight is smoking by pregnant women. The first report on the increased risk of fetal hypotrophy in smoking women was published in 1957, and to this day, studies are being carried out with the aim of explaining the changes in the mother as well as the child provoked by tobacco smoke [9]. Newborns of smoking mothers weigh an average of 88–450 g less at birth than newborns of non-smoking mothers, and the underweight grows proportionally to the number of cigarettes smoked [10,11,12,13,14,15,16,17]. According to Grjibovski et al. [17] and Lockhart et al. [14], birth weight decreased 27–41 g per cigarette smoked per day during pregnancy. A Brazilian cross-sectional study indicated that birth weight in infants whose mothers smoked 6 to 10 cigarettes per day was 320 g lower and 435 g in infants whose mother smoked 11–40 cigarettes per day compared with infants born to non-smoking mothers [16]. It is also known that children born to mothers exposed to second hand smoke during pregnancy are 40–60 g smaller than children of women without exposure to tobacco smoke [18].

By damaging the structure and disrupting the function of the placenta, toxic components of tobacco smoke impair gas exchange and the active transport of nutrients essential for the fetus. The disturbed glucose flow observed in smokers may result in a disruption of carbohydrate metabolism and decreased insulin levels and, consequently, a slowdown in fetal development [2,19].

Despite extensive knowledge about the structure and role of adiponectin and insulin growth factor system markers in the body, their interaction and role in regulating fetal and infant growth are still less known, especially when exposed to tobacco smoke in utero.

This is particularly important because, although there have been many educational programs, the exposure to tobacco smoke of newborns and children is still very high [20]. In Poland, around 15% of women smoke cigarettes during pregnancy; 30% of non-smoking pregnant women are exposed to ETS in the home and work environment, and more than 40% of children are exposed to passive inhalation of cigarette smoke due to parental smoking [21]. Children of smoking mothers have a higher risk of low birth weight and future risk of developing metabolic disorders, cardiovascular diseases, reduced psychomotor skills, and behavioral problems [18,22].

Therefore, the aims of this study were: (1) to evaluate the potential influence of maternal smoking during pregnancy on the studied biochemical parameters; (2) to determine the relationship between total adiponectin, HMW adiponectin, selected somatomedins, and the birth weight of newborns of smoking and non-smoking mothers.

## 2. Materials and Methods

### 2.1. Participants

All participating mothers were informed of the nature and purpose of the study and written consent was provided for blood sample analysis and linking the results to the data collected form questionnaires. The study conforms to the principles outlined in the Declaration of Helsinki and was approved by the Ethical Committee of the Institute of Mother and Child (Decision No. 9/2010).

We examined 78 mother–child pairs recruited between June 2012 and December 2014 on the day of their admission for delivery at the Institute of Mother and Child in Warsaw. We enrolled 41 pregnant women (admitted consecutively) who smoked during pregnancy as a study group and 37 tobacco abstinent pregnant women of a similar age and age of gestation as a control group. All pregnant women participating in the study had structurally normal, uncomplicated single pregnancies and delivered at term healthy neonates with a weight appropriate for the gestational age (confirmed by ultrasound before 20 weeks of gestation).

The exclusion criteria included unreliable gestational age (under 37 weeks), multiple gestation, preeclampsia, chronic hypertension, diabetes mellitus, active hepatitis, renal and cardiovascular diseases, infection, and infertility treatment. All the subjects lived in an urban area and remained on a mixed diet; none of the mothers reported drinking alcohol or using drugs or illicit substances, and none of the fetuses showed abnormalities.

At the recruitment visit, smoking history was obtained using a short standard questionnaire designed for the study. The study group included women who smoked at least two years before conception and continued smoking at least five cigarettes per day during the entire pregnancy. Non-smokers were defined as those women who had never smoked and were not exposed to environmental tobacco smoke during their pregnancy (smoking spouse or co-workers). The control group included women who never smoked and were not exposed to environmental tobacco smoke during the pregnancy both at home and in the work place. To confirm the group classification, serum cotinine levels (the main nicotine metabolite) were determined in all patients. To separate smokers from non-smokers a cut-off value of ≥13.7 µg/L was used [23].

We also collected data from the patients’ histories concerning the course of pregnancy, gestational age (GA), mode of delivery (vaginal versus caesarean section), sex, birth weight, length, Apgar scores, and neonate condition.

Pre-pregnancy body max index (BMI) was calculated according to the formula: pre-pregnancy weight (kg)/height (m^2^). Newborn infants were evaluated in the first 24 h of life. Neonatal length and weight were determined using a measuring board to the nearest 0.1 cm and a calibrated scale to the nearest 10 g.

### 2.2. Blood Sampling and Biochemical Analysis

As previously described, maternal peripheral blood samples (5 mL) were taken on the day of admission for delivery while performing routine examination [24]. Mixed venous and arterial blood samples (5 mL) were collected at the time of delivery from the umbilical vein before placental separation. In order to obtain the serum, the blood was centrifuged at 2500× *g*, at 4 °C for 10 min and was stored in small portions for subsequent biochemical analysis.

Cotinine levels in the serum were evaluated by immunoenzymatic method using a commercially available kit (Cotinine one-step ELISA, Calbiotech Inc., Spring Valley, CA, USA). The inter- and intra-assay coefficients of variability were found in less than 10%. The sensitivity limit was 1.0 ng/mL.

Serum total adiponectin and its multimeric form levels were determined by the multimeric adiponectin enzyme-linked immunosorbent assay (ELISA, ALPCO Diagnostics, Salem, NH, USA). Adiponectin multimers were selectively measured after sample pretreatment with two proteases that specifically digested the trimeric forms or both the hexameric and trimeric forms. In this assay, total adiponectin and HMW concentrations were determined directly, while LMW and MMW levels were calculated indirectly. MMW concentration was calculated by subtracting the combined concentration of MMW + HMW from the total adiponectin. The inter- and intra-assay coefficients of variability were found less than 5.0% and 5.3% for total adiponectin, 6.0% and 4.1% for MMW + HMW, 5.7% and 3.3% for HMW isoform, respectively. Assay sensitivity was 0.019 ng/mL. We calculated the ratio of HMW/total adiponectin levels (S_A_) for maternal and cord blood adiponectin concentrations.

IGF-I, IFBP-1, and IGFBP-2 values were measured by immunoassay (ELISA, DRG, Marburg Mediagnost, Reutlingen, Germany). The analytical sensitivity of the assay was 1.29 ng/mL for IGF-I, 0.02 ng/mL for IGFBP-1, and 0.2 ng/mL for IGFBP-2, respectively. The inter- and intra-assay coefficients of variation were less than 7.2% and 4.7% for IGF-I, 7.4% and 6.8% for IGFBP-1, and 10% for IGFBP-2, respectively.

### 2.3. Statistical Analysis

Statistical analyses were carried out with the SPSS statistical package version 17.1 (SPSS INC., Chicago, IL, USA). Before statistical analysis, the normality of data distribution in the groups was checked using the Kolmogorov–Smirnov test. The normally distributed data were expressed as means and standard deviation (SD) and the non-normally distributed data as medians and interquartile range (25–75th percentiles). Either the Student *t*-test or the Mann–Whitney *U* test was used to compare the baseline characteristics and biochemical parameters. Similarly, a correlation analysis was performed based on Pearson or Spearman coefficients, adequately to the distribution of variables. Chi squared test was used to test the associations between categorical variables. Multivariate regression models with total and HMW adiponectin as the dependent variables were performed to examine the potential impact of the other tested parameters. Moreover, the influence of the studied biochemical markers on birth weight was estimated using a linear regression model. The values of β standardized regression coefficients and B unstandardized regression coefficients with corresponding 95% confidence intervals were presented in the tables, together with a change in R-squared coefficients after each variable was entered. The models were estimated for the whole group and separately for smokers and non-smokers. A level of statistical significance was set a *p*-value equal or less than 0.05.

## 3. Results

Clinical characteristics and biochemical measurements of the 78 participants and their newborns are presented in Table 1. The characteristics are stratified based on maternal tobacco smoking status as non-smoking and smoking group. Both studied group were comparable (except for cigarette smoking habits) without severe complications during delivery. In the non-smoking group there was one case of an emergency cesarean section due to a large baby weight and in the smoking group due to abnormal presentation. Mean birth weight and body length of the smoking mothers’ infants were significantly lower (*p =* 0.000; *p* = 0.007, respectively) compared with the abstinent group. The Apgar score was similar in the two groups.

Total maternal adiponectin and its HMW form levels as well as S_A_ were lower in the smoking group compared with the tobacco abstinents, whereas no differences were observed in the remaining multimeric forms, such as MMW and LMW. In the smoking mothers, serum concentrations of IGF-I were significantly lower, IGFBP-1 significantly higher, and IGFBP-2 did not differ from the non-smoking group. Similar results were observed in the case of neonate cord blood.

In smoking mothers, total and HMW adiponectin concentrations correlated negatively with cotinine levels. Cord serum HMW adiponectin concentrations were inversely associated with the number of cigarettes/day and with cotinine levels while total adiponectin only with the number of cigarettes/day. A negative relation between S_A_ values and cotinine in cord blood also was found. In both groups, mother and child, IGF-I levels correlated negatively whereas IGFBP-1 positively with the number of cigarettes smoked per day. Smoking was not associated with serum IGFBP-2, MMW and LMW concentrations in maternal and umbilical cord blood (Table 2).

Multivariate analysis performed in the whole group of mothers confirmed the highest impact of smoking on total and HMW adiponectin concentration. In this model, cotinine and IGFBP-2 explained 27.5% of the total adiponectin variation while cotinine and cigarettes/day 33.8% of the HMW form variation. In the model of cord blood, cotinine was of significant importance for the HMW adiponectin concentration (R-squared was 32.5%) (Table 3).

Spearman correlation analysis, estimated separately for smokers and non-smokers (Table 4), showed that neonatal birth weight was positively correlated with total and HMW adiponectin concentrations in umbilical cord blood. Birth weight was also inversely associated with IGFBP-1 and positively correlated with IGF-I level in maternal serum as well as in cord blood. MMW, LMW adiponectin, and IGFBP-2 concentrations were not significantly associated with any studied markers determining the intensity of smoking.

In multivariate analysis estimated for the total group (smoking and non-smoking individuals), independent predictors of neonate birth weight were identified (Table 5). In addition to maternal smoking habit, IGF-I and IGFBP-1 concentrations appeared to be an important predictor of birth weight. Based on R-squared coefficient, this model explained 42.7% of the variability in birth weight in the case of maternal blood and 56.0% in the case of umbilical cord blood parameters.

## 4. Discussion

The present study showed that fetal exposure to tobacco smoke in utero may lead to changes in the secretion of adiponectin isoforms and insulin-dependent growth factors, which may consequently lead to a reduction in the birth weight of the child. Maternal smoking during pregnancy is a well-known cause of low birth weight in infants and has been associated with an increased risk of adiposity, insulin resistance, and type 2 diabetes in children and adults [14,18]. There are several mechanisms that may explain the pathophysiological relations between smoking and adiponectin downregulation. Increased oxidative stress and chronic inflammatory processes observed in smokers are the main cause of endothelial dysfunction and may lead to a decrease in circulating adiponectin due to their increased consumption by damaged vascular walls [25,26]. In response to nicotine and free radicals, endothelial cells secrete many cytokines, including TNF-α (tumor necrosis factor-α), that reduce the expression and secretion of adiponectin [19]. There are also studies confirming the direct effect of nicotine on the inhibition of adiponectin gene expression in mouse adipocytes [25].

Despite the well-known inverse association between smoking and adiponectin levels in current smokers, there have been conflicting reports on the effects of smoking on this protein concentration in the serum of pregnant women and cord blood [25,26,27,28]. We confirmed results of other studies in which infants and mothers from a smoking group had lower total adiponectin concentrations compared with a tobacco abstinent group [26,28]. A similar level of adiponectin was observed in smokers and non-smokers by Fleisch et al. [27]; however, the authors conducted the study in a group of mothers who stopped smoking in the first trimester of pregnancy, while it has been documented that smoking cessation improves adiponectin status [25,29,30]. The lack of differences in the study by Fang et al. [15] may result from the small research group and relatively low exposure, when smokers decrease the level of adiponectin in a dose-dependent manner [13,24].

There are few human studies assessing adiponectin isoforms in the serum of non-smoking pregnant women and cord blood [1,2,3,4]. To the best of our knowledge, this is the first report demonstrating and comparing adiponectin isoform levels in smoking and non-smoking mother-child pairs. We found lower levels of HMW adiponectin and S_A_ index in the smoking mothers and the cord blood of their children than in the non-smoking group. The levels of the other adiponectin isoforms did not differ between both study groups. Our results confirmed the observations of Catalano et al. [31] that hypoadiponectinemia during pregnancy is reflected in a smaller amount of high molecular weight multimers. Similarly to Odden et al. [1], we showed that the HMW adiponectin is a dominant form also in newborns. We confirmed our previous results regarding the close correlation between smoking intensity and a decrease in total adiponectin level and also showed a predictive effect of cotinine on HMW fraction concentration in the serum of the mother and the newborn [24]. Due to the anti-inflammatory and antioxidant properties of HMW, its decrease in smokers may be due to the prevention of damage associated with the toxic effects of tobacco smoke.

The relationship between adiponectin and birth weight has been studied for many years, but the results are inconclusive, and there is no study on pregnant smokers [21,32,33]. We found that the birth weight of newborns was positively associated with total and HMW adiponectin concentrations in the umbilical cord but not in the mother’s blood in the smokers and the non-smokers group. Similarly to our study group of smokers, lower levels of adiponectin multimers in maternal serum were observed in mothers who gave birth to a small-for-gestational age child. This study also did not confirm a significant relationship between maternal parameters and birth weight [34]. In contrast to maternal concentrations, neonatal concentrations of total and HMW adiponectin positively correlated with birth weight [35]. Maternal adiponectin did not cross the placenta, but many fetal tissues can produce adiponectin, which contributes to high fetal concentration and fetal development [19,36]. The direct effect of adiponectin on tissue sensitivity to insulin and IGF system components can explain the positive effect associated between adiponectin and birth weight [37].

Insulin is the primary regulator of glucose metabolism, but the IGF axis also might play a role in maintaining glucose homeostasis. The high, proportional relationship between the anthropometric features of the newborn and the level of IGF-I in umbilical cord blood observed by other authors suggests a significant effect of this factor on fetal development and weight [38,39]. It is assumed that IGF-1 inhibits lipolysis, gluconeogenesis, increases glucose transport to adipocytes, and reduces the release of free fatty acids [40]. IGFBP-1, which is the main regulator of somatomedins during pregnancy, is synthesized in the placenta, and its high concentrations are found in the amniotic fluid [7]. The concentration of IGFBP-1 in the blood is regulated by insulin availability. IGFBP-2 is also regulated by changes in nutritional status, and it is speculated that IGFBP-1 and IGFBP-2 could be able to modulate insulin sensitivity in an IGF-independent manner [5,41]. The IFFBP-1 level increases as pregnancy progresses similar to IGF-I as opposed to decreased IGFBP-2 concentrations (due to an increase in IGFBP-proteases) [4]. Boyne et al. [6] observed that the birth weight of newborns was directly proportional to IGF-I concentration and inversely to the concentration of IGFBP-1 in maternal serum in the third trimester of pregnancy and in umbilical cord blood. Additionally, intrauterine growth retardation is associated with a decrease in IGF-I and an increase in IGFBP-1 and IGFBP-2 levels in fetal blood [42]. In agreement with our previous study and the studies of other authors, we confirmed the negative effect of smoking on maternal and cord blood IGF-I concentrations [15,27,43,44]. Our study is the first to present IGFBP-1 and IGFBP-2 in smoking matched maternal pairs. We found significantly higher concentrations of IGFBP-1 in the smoking group of mothers and children, whereas IGFBP-2 did not differ in both groups. The decreased IGF-I level co-existing with elevated IGFBP-1 level in the smoking mother group may reflect malnutrition and can indicate a low metabolic rate in their offspring. We postulated that in addition to mother’s smoking habit, IGF-I and IGFBP-1 concentrations appeared to be an important predictor of infant birth weight.

There are many possible mechanisms explaining the effects of smoking on the growth-promoting IGF axis in utero. Components of cigarette smoke induce increased secretion of insulin-counter regulatory hormones, such as cortisol and catecholamine, leading to intensified lipolysis processes and free fatty acid formation, resulting in elevated levels of lipid peroxidation products [25,45]. Increased oxidative stress and chronic inflammatory processes in women who smoke tobacco during pregnancy are the main cause of endothelial dysfunction in the utero-placental compartment, which may in turn lead to fetal hypoxia. An increase in pro-inflammatory cytokine levels accompanied by a smoking-related chronic inflammatory state may also contribute to an increase in IGFBP-1 as well as a reduction in total IGF-I concentration [41]. Moreover, the relationship between low birth weight and increased IGFBP-1 concentration in umbilical cord blood coexists with a degree of fetal hypoxia [46]. It is also known that functional nicotinic receptors are present in pancreatic islets and β cells, and nicotine exposure can cause dysfunction and increased β cell apoptosis [47]. The adverse effect of tobacco on insulin growth factor and β cell function in human fetuses and newborns has been confirmed by Fang et al. [15]. Finally, disturbances in the insulin-like growth factor system could be associated with a lower placental growth factor (GH) concentration observed by other authors in smokers [48].

We acknowledge several potential limitations of our study. First, only healthy Caucasian women living in a large urban and surrounding area took part in our study, so we cannot extrapolate the results to all pregnant women populations. Additionally, we cannot achieve a percentage of smokers representative of a normal pregnant population with the enrolment method we used. The applied research scheme is not typical for a case-control study with retrospective assumptions [49]. This is a prospective comparison of cohorts exposed and not exposed to the effect of one factor, in this case, tobacco smoking. The next limitation was the relatively small sample size resulting from the limited availability of umbilical cord blood. However, the compared groups were homogenous and did not differ in terms of their size and basic characteristics. We were unable to measure serum insulin and glucose levels, but it was clear from the medical history that all of participants were routinely screened for gestational diabetes mellitus (GDM), and there were no complications during pregnancy. Moreover, we did not have any data on serum lipids, but it has been known for a long time that smoking during pregnancy markedly affects maternal and fetal lipid metabolism [50]. However, the ability to assess the selected biochemical parameters in a group of women who smoke an average of 10 cigarettes per day throughout a pregnancy and in their offspring is an advantage that can compensate for the above limitations.

## 5. Conclusions

Our findings showed that levels of total and HMW adiponectin in umbilical cord blood may have a significant effect on fetal development. Both IGF-I and IGFBP-1 concentrations also play an essential role in fetal growth as an important predictor of birth weight. Cigarette smoking during pregnancy negatively affects the adiponectin and insulin growth factor profile in the serum of women and cord blood and may be the reason for the lower birth weight of smokers newborns compared with nonsmokers offspring.

## Figures and Tables

**Table 1:** Clinical characteristics and biochemical measurements in smoking and non-smoking matched maternal cord pairs (*n* = 78).

Characteristic	Smoking *n* = 41	Non-Smoking *n* = 37	*p*-Value
Maternal age (years)	28.2 ± 4.4	29.5 ± 4.6	0.218
Ethnic origin: Caucasian (%)	100	100	-
Maternal weight (kg)	63.9 ± 5.2	65.3 ± 5.4	0.230
Maternal height (cm)	165.3 ± 0.5	165.0 ± 0.4	0.630
Pre-gravid Body Mass Index (kg/m^2^)	23.6 ± 1.1	24.2 ± 1.2	0.081
Gestational age of delivery (weeks)	39 (39–40)	39 (39–40)	0.103
Delivery: Cesarean/Vaginal (*n*)	1/40	1/36	-
Neonatal gender Female/Male (*n*)	19/22	16/21	0.213
Birth weight (g)	3131.7 ± 416.4	3531.6 ± 459.1	0.000
Birth body length (cm)	54.4 ± 2.8	56.1 ± 2.5	0.007
Apgar score (5th min)	10 (9–10)	10 (10–10)	0.576
Number of cigarettes/day	10 (5–15)	0	-
Time of smoking before conception (year)	8 (5–10)	0	-
Cotinine (µg/L)	91.1 (67.1–108.5)	0	-
Pregnant women			
Adiponectin (μg/mL)	5.8 ± 2.4	7.4 ± 3.2	0.013
HMW adiponectin (μg/mL)	3.2 ± 1.7	4.8 ± 3.0	0.006
S_A_ (%)	53.6 ± 13.1	62.9 ± 12.4	0.002
MMW adiponectin (μg/mL)	1.14 ± 0.80	1.26 ± 0.77	0.481
LMW adiponectin(μg/mL)	1.3 (0.8–1.9)	1.2 (0.6–1.7)	0.704
IGF-I (ng/mL)	328.6 ± 129.3	399.1 ± 117.7	0.014
IGFBP-1 (ng/mL)	191.2 ± 97.9	153.0 ± 62.7	0.042
IGFBP-2 (ng/mL)	163.8 (126.6–232.6)	127.8 (101.3–190.0)	0.117
Umbilical cord blood			
Adiponectin (μg/mL)	18.6 ± 4.8	21.6 ± 7.9	0.001
HMWadiponectin (μg/mL)	12.8 ± 4.7	17.6 ± 7.3	0.047
S_A_ (%)	68.7 ± 15.3	80.2 ± 9.5	0.000
MMW adiponectin (μg/mL)	2.5 (1.0–3.2)	1.20 (0.5–2.6)	0.061
LMW adiponectin (μg/mL)	2.3 (1.3–5.1)	1.70 (1.3–2.5)	0.064
IGF-I (ng/mL)	61.3 ± 41.8	87.7 ± 45.0	0.009
IGFBP-1 (ng/mL)	90.9 ± 37.9	75.4 ± 26.6	0.039
IGFBP-2 (ng/mL)	1148.3 ± 414.5	1091.5 ± 418.3	0.549

Values are means ± standard deviation (SD). Values are median and interquartile range (25th–75th percentiles). HMW—high molecular weight; S_A_—HMW/total adiponectin ratio; MMW—middle molecular weight; LMW—low molecular weight; IGF-I—insulin-like growth factor I; IGFBP-1—insulin-like growth factor binding protein 1; IGFBP-2—insulin-like growth factor binding protein 2.

**Table 2:** Relationships between levels of studied biochemical parameters and cotinine as well as number of cigarettes/day.

Biochemical Parameters	Cotinine				Number of Cigarettes/Day			
	Maternal		Umbilical Cord Blood		Maternal		Umbilical Cord Blood	
	*r*	*p*-Value	*r*	*p*-Value	*r*	*p*-Value	*r*	*p*-Value
Adiponectin	−0.388	0.012	−0.286	0.070	−0.102	0.526	−0.363	0.020
HMW adiponectin	−0.409	0.008	−0.447	0.003	−0.093	0.565	−0.413	0.007
S_A_	−0.260	0.100	−0.390	0.012	−0.044	0.784	−0.262	0.098
MMW adiponectin	−0.272	0.170	0.023	0.886	−0.130	0.418	0.055	0.735
LMW adiponectin	0.038	0.815	0.133	0.408	0.010	0.951	−0.080	0.621
IGF-I	−0.246	0.121	−0.204	0.200	−0.467	0.002	−0.348	0.026
IGFBP-1	0.129	0.422	0.299	0.058	0.389	0.012	0.363	0.020
IGFBP-2	0.029	0.855	0.030	0.852	0.202	0.206	−0.007	0.936

HMW—high molecular weight; S_A_—HMW/total adiponectin ratio; MMW—middle molecular weight; LMW—low molecular weight; IGF-I—insulin-like growth factor I; IGFBP-1—insulin-like growth factor binding protein 1; IGFBP-2—insulin-like growth factor binding protein 2.

**Table 3:** Multivariate regression models for total adiponectin and its HMW isoform in pregnant women and umbilical cord blood.

Biochemical Parameters and Tobacco Smoking Markers	Adiponectin			HMW Adiponectin		
	B	95% CI	*p*-Value	B	95% CI	*p*-Value
Pregnant women ^1^	R^2^ = 0.275		0.012	R^2^ = 0.338		0.001
Cotinine (µg/L)	−0.059	−0.103/−0.015	0.009	−0.016	−0.025/−0.007	0.000
Number of cigarettes/day	0.247	−0.083/0.577	0.140	0.073	0.007/0.138	0.029
Time of smoking before conception (year)	0.028	−0.229/0.285	0.829	0.007	−0.044/0.058	0.789
IGF-I (ng/mL)	0.003	−0.003/0.009	0.293	0.000	−0.001/0.001	0.691
IGFBP-1 (ng/mL)	−0.002	−0.011/0.008	0.715	−0.001	−0.003/0.001	0.289
IGFBP-2 (ng/mL)	0.009	0.001/0.017	0.026	0.001	−0.001/0.003	0.180
Umbilical cord blood ^2^	R^2^ = 0.299		0.009	R^2^ = 0.323		0.004
Cotinine (µg/L)	−0.054	−0.159/0.051	0.309	−0.009	−0.017/0.000	0.040
Number of cigarettes/day	0.168	−0.563/0.898	0.648	0.033	−0.025/0.092	0.260
Time of smoking before conception (year)	0.443	−0.141/1.027	0.135	0.043	−0.004/0.090	0.071
IGF-I (ng/mL)	−0.003	−0.048/0.042	0.886	−0.001	−0.005/0.003	0.585
IGFBP-1 (ng/mL)	0.045	−0.011/0.100	0.111	0.002	−0.002/0.007	0.355
IGFBP-2 (ng/mL)	−0.002	−0.006/0.001	0.219	0.000	0.000/0.000	0.304

^1^ Variables for adjusted analysis: maternal age, pre-gravid BMI, fetal gender, smoking status (no = 0/yes = 1). ^2^ Variables for adjusted analysis: delivery age, birth weight, birth body length, fetal gender, smoking status (no = 0/yes = 1). HMW—high molecular weight; IGF-I—insulin-like growth factor I; IGFBP-1—insulin-like growth factor binding protein 1; IGFBP-2—insulin-like growth factor binding protein 2.

**Table 4:** Linear relationship between birth weight and studied biochemical parameter levels.

Biochemical Parameters	Maternal				Umbilical Cord Blood			
	Smoking Group		Non-Smoking Group		Smoking Group		Non-Smoking Group	
	*r*	*p*-Value	*r*	*p*-Value	*r*	*p*-Value	*r*	*p*-Value
Adiponectin	0.134	0.403	0.149	0.378	0.436	0.004	0.422	0.009
HMW adiponectin	0.055	0.731	0.167	0.324	0.397	0.010	0.408	0.012
S_A_	−0.063	0.695	0.007	0.969	0.200	0.210	0.105	0.538
IGF-I	0.451	0.003	0.444	0.006	0.536	0.000	0.481	0.003
MMW adiponectin	0.294	0.062	0.022	0.896	0.160	0.317	0.259	0.122
LMW adiponectin	−0.004	0.982	0.003	0.985	0.130	0.416	0.032	0.853
IGFBP-1	−0.397	0.010	−0.600	0.000	−0.519	0.001	−0.496	0.002
IGFBP-2	−0.057	0.722	−0.060	0.724	0.044	0.786	−0.085	0.616

HMW—high molecular weight; S_A_—HMW/total adiponectin ratio; MMW—middle molecular weight; LMW—low molecular weight; IGF-I—insulin-like growth factor I; IGFBP-1—insulin-like growth factor binding protein 1; IGFBP-2—insulin-like growth factor binding protein 2.

**Table 5:** Estimated β-coefficients for the studied biochemical parameter levels in multivariate linear regression models explaining birth weight variability in the studied matched maternal-cord pairs.

Biochemical Parameters	Maternal		Umbilical Cord Blood	
	β	*p*-Value	β	*p*-Value
Adiponectin	−0.117	0.717	0.445	0.256
HMW adiponectin	0.323	0.413	−0.153	0.751
S_A_	−0.226	0.203	0.156	0.478
IGF-I	0.245	0.031	0.271	0.009
IGFBP-1	−0.317	0.005	−0.294	0.004
IGFBP-2	0.008	0.931	0.027	0.743
Smoking status (no = 0; yes = 1)	−0.293	0.006	−0.155	0.096
	R^2^ = 0.427	*p* = 0.000	R^2^ = 0.560	*p* = 0.000

HMW—high molecular weight; S_A_—HMW/total adiponectin ratio; IGF-I—insulin-like growth factor I; IGFBP-1—insulin-like growth factor binding protein 1; IGFBP-2—insulin-like growth factor binding protein 2.
